# Decision-making inflexibility in a reversal learning task is associated with severity of problem gambling symptoms but not with a diagnosis of substance use disorder

**DOI:** 10.1186/s40359-020-00482-6

**Published:** 2020-11-10

**Authors:** María F. Jara-Rizzo, Juan F. Navas, Jose A. Rodas, José C. Perales

**Affiliations:** 1 Faculty of Psychology, University of Guayaquil, Guayaquil, Ecuador; 2 Department of Clinical Psychology, Complutense University of Madrid, Madrid, Spain; 3 School of Psychology, University College Dublin, Dublin, Ireland; 4 Department of Experimental Psychology; Mind, Brain and Behavior Research Centre (CIMCYC), University of Granada, Granada, Spain

**Keywords:** Addictive disorders, Gambling disorder, Gambling severity, Compulsivity, Learning inflexibility, Probabilistic reversal learning task

## Abstract

**Background:**

Decisions made by individuals with disordered gambling are markedly inflexible. However, whether anomalies in learning from feedback are gambling-specific, or extend beyond gambling contexts, remains an open question. More generally, addictive disorders—including gambling disorder—have been proposed to be facilitated by individual differences in feedback-driven decision-making inflexibility, which has been studied in the lab with the Probabilistic Reversal Learning Task (PRLT). In this task, participants are first asked to learn which of two choice options is more advantageous, on the basis of trial-by-trial feedback, but, once preferences are established, reward contingencies are reversed, so that the advantageous option becomes disadvantageous and vice versa. Inflexibility is revealed by a less effective reacquisition of preferences after reversal, which can be distinguished from more generalized learning deficits.

**Methods:**

In the present study, we compared PRLT performance across two groups of 25 treatment-seeking patients diagnosed with an addictive disorder and who reported gambling problems, and 25 matched controls [18 Males/7 Females in both groups, M_age_(SD_age_) = 25.24 (8.42) and 24.96 (7.90), for patients and controls, respectively]. Beyond testing for differences in the shape of PRLT learning curves across groups, the specific effect of problematic gambling symptoms’ severity was also assessed independently of group assignment. In order to surpass previous methodological problems, full acquisition and reacquisition curves were fitted using generalized mixed-effect models.

**Results:**

Results showed that (1) controls did not significantly differ from patients in global PRLT performance nor showed specific signs of decision-making inflexibility; and (2) regardless of whether group affiliation was controlled for or not, gambling severity was specifically associated with more inefficient learning in phases with reversed contingencies.

**Conclusion:**

Decision-making inflexibility, as revealed by difficulty to reacquire decisional preferences based on feedback after contingency reversals, seems to be associated with gambling problems, but not necessarily with a substance-use disorder diagnosis. This result aligns with gambling disorder models in which domain-general compulsivity is linked to vulnerability to develop gambling-specific problems with exposure to gambling opportunities.

## Background

Balanced decision-making is crucial for adaptive daily functioning. Consequently, anomalies of decision-making processes are present in a range of psychopathological conditions [1–4]. In more direct relation to the aims of the present study, addiction has been described as a disorder of the ability to make good decisions, namely to make choices regarding the potentially addictive behavior (using drugs, gambling) that overcome impulses, for the sake of more beneficial long-term goals [5–7]. Individuals suffering from an addictive disorder thus persist in a harmful behavior in spite of its negative consequences (e.g. economic losses, health, family and work problems) (DSM-5 [8]). In concordance with this observation, a number of studies have tried to test the prediction that addicted patients present domain-general decision-making alterations that could predate the disorder onset, contribute to its chronicity or complication, or occur as a consequence of the addictive process itself [9–11].

Among the aspects of decision making that are relevant to understand addictive processes is behavioral flexibility, namely the ability to readjust preferences in response to reward contingency changes in decision-making-under-ambiguity tasks. Inflexibility would be manifested in a transient inability to stop choosing a given option that is no longer advantageous (perseveration), or, more generally, in a difficulty to relearn action-outcome contingencies that depart from the ones that were learnt in the initial acquisition phase.

The relevance of decision-making inflexibility for addictive behaviors stems from the fact that it is hypothesized to reflect domain-general proneness to compulsivity [12, 13], which, in turn, has been theorized to be a transdiagnostic vulnerability factor for addictive disorders and other psychopathologies [14, 15]. In different theories, (a) addictive behaviors transition from being goal-driven to being stimuli or context-driven (habitual/compulsive, [16, 17]), or, alternatively, (b) addiction-related rewards acquire a disproportionate motivational value [18]. Independently of which of these approaches is correct, it seems obvious that being unable to fine-tune the associations between decision options and outcomes, or to behave accordingly, would render the individual more vulnerable to the progression of loss of control over potentially addictive behaviors.

There are several ways to operationalize decision-making inflexibility in the laboratory [19, 20]. However, none of the available protocols is sensitive only to inflexibility. Crucially, reacquisition after contingency change is inextricably linked to more general contingency learning differences, i.e. any individual differences in acquisition will contaminate gross reacquisition differences. So, detecting inflexibility previously requires experimentally dissociating acquisition learning and artifact-free (in)flexibility (for a detailed discussion on this matter and its methodological subtleties, see [21]).

This distinction is not only of methodological importance. There is some consensus that acquisition learning is computationally simpler than adjustment to unsignaled contingency changes. Extinction, for instance, is not just the vanishment of previous conditioning, but a context-dependent learning process about the omission of the reinforcer [22]. Similarly, reversal learning seems to require high-order mechanisms to restructure the set of learned associations (for an updated view, see [23]). So, once the dissociation is granted, computational modelling is required to identify the cognitive processes originating the two parts of such a dissociation [21]. Although the present study concerns only the first stage of this process, the constraints it imposes on computational modelling will be sketched in the discussion section.

### The probabilistic learning task and problematic gambling

The most pervasively used task to investigate decision-making inflexibility in the lab is the Probabilistic Reversal Learning Task (henceforth, PRLT). In each trial of this task, two choice options are presented to the learner, one advantageous (more likely to ensue reward; e.g. virtual points or money), and the other disadvantageous (more likely to ensue some kind of punishment). Initially, the individual has no other possibility than making her decision at random, but her choices grow attuned to reward and punishment contingencies as the task progresses. However, at some point, and without prior notice, the contingencies are reversed, and the individual needs to update her preferences on the basis of the new contingencies.

As noted above, decision-making inflexibility can be used as an individual-differences measure of compulsivity [24, 25]. Unfortunately, most previous attempts to compare PRLT performance across groups of individuals with and without addictive behaviors are not free of methodological and interpretational problems. In general, there is no unanimity regarding the best way to measure inflexibility in the PRLT. In a recent meta-analysis and systematic review, van Timmeren et al. [19], found that the studies that used the PRLT do not reveal significant levels of behavioral inflexibility in individuals with gambling disorder. However, this could be due to the diversity of procedures and measures used to operationalize PRLT performance. Different studies used, for instance, the amount of money or points earned [26, 27], the number of correct choices [28–30] or the number of consecutive errors after each reversion (i.e. perseverative errors, [31, 32]).

In the present work, we will follow Perandrés-Gómez et al.’s [21] approach to analyze full acquisition and re-acquisition curves in a PRLT with four phases: one in which preferences are first established, and three more resulting from two contingency reversals [33]. The first aim was to determine whether group affiliation (patients with a substance use disorder with symptoms of comorbid problematic gambling vs healthy controls) has any effect on the form of learning curves in each phase, or their variation across phases. Inflexibility can be corroborated by detecting any reacquisition disadvantage in phases with reversed contingencies (2 and 4), relative the ones with contingencies in the original direction (1 and 3) that is experimentally dissociable from global differences in task performance (e.g. phase-independent learning rates or asymptotes). More importantly, we will test whether the effect of contingency reversal depends itself on group (i.e. whether patients show more signs of decision-making inflexibility than controls). Subsequently, we will specifically analyze participants’ performance in relation to the severity of their problematic gambling symptoms, regardless of (or controlling for) group affiliation. By assessing the relationship between gambling severity and PRLT performance, independently of group, we intended to dissociate the effect of problematic gambling from the one of other addictive behaviors.

Decision-making inflexibility in reversed contingency phases of the PRLT has been previously reported to be associated with disordered gambling [21, 29]. This proneness towards compulsivity would explain to some extent the easiness with which disordered gamblers adhere to an initially favorable reinforcement contingency, but are later incapable of abandoning it [34–36]. Therefore, from this perspective, PRLT inflexibility would be expected to be linked to higher severity of disordered gambling symptoms, independently of the diagnosis of other addictive disorders.

## Methods

### Participants

Twenty-five patients under treatment for an addictive disorder were recruited from the centers *Centro de Recuperación Nueva Luz* and *Centro de Recuperación Integral de Alcoholismo y Drogadicción* (CRIAD), from Guayaquil, Ecuador. Convenience sampling was used to recruit 25 healthy controls, as closely matched as possible with patients on relevant covariates. Some of the control participants were contacted using announcements in the School of Psychology of the University of Guayaquil, and others were recruited among acquaintances of the patients.

All patients were under treatment for at least one addictive disorder (most of them, for alcohol use disorder), and were diagnosed with the DSM-IV-TR diagnostic criteria. The inclusion criteria for both groups were: (1) being between 18 and 65 years old, and (2) no history of head trauma or neurological problems, and not to be diagnosed with any psychiatric or psychological disorder (apart from the addictive disorder in the group of patients). Patients were included in the sample and considered for further assessments and analyses only if they informed of a previous history of significant problems as a consequence of gambling. Severity of gambling symptoms was assessed with the South Oaks Gambling Screen (SOGS, Spanish version; [37]). Nineteen of the 25 patients actually scored above the SOGS threshold for gambling disorder. The 6 patients who informed to have suffered gambling problems in the past but did not meet the criteria for current gambling disorder were kept in the study sample. The sociodemographic and clinical profile of each group is reported in Table 1.

**Table 1:** Sociodemographic and clinical features: means, standard deviations, and Bayes factors, expressing support for the alternative hypothesis

	Group	Mean	SD	BF_10_
Age	HC	24.96	7.908	0.303
	Patients	25.24	8.428	
Education	HC	14.33	3.131	4.52
	Patients	12.36	2.307	
Income	HC	4.21	1.607	0.317
	Patients	4.04	1.695	
SOGS	HC	0.44	1.08	106,137
	Patients	7.72	4.61	
Alcohol misuse	HC	0.12	0.18	1274
	Patients	0.63	0.34	
Drug misuse	HC	0.02	0.10	66,121
	Patients	0.77	0.25	

HC, healthy controls; SOGS, South Oaks Gambling Screen

### Procedure

Each participant was assessed in a single session lasting ~ 2 h. Patients were assessed in the rehabilitation clinics, and control participants in the premises of the School of Psychology of the University of Guayaquil. All the assessments were performed by an Ecuadorian clinical psychologist with a master’s degree in neuroscience. The assessment protocol was divided into four blocks (cognitive tests, computer tasks, paper-and-pencil emotion and personality tests, and a clinical interview). The order of blocks and tasks within each block were counterbalanced for all participants. The instruments used were the Wechsler Intelligence Scale for Adults-III (WAIS-III: vocabulary and matrices [38]), an impulsive behavior scale (UPPS-P [39]), the Sensitivity to Punishment and Sensitivity to Reward Questionnaire (SPSRQ-20 [40]), the South Oaks Gambling Screen (SOGS [38]), the MultiCAGE [41], and the Probabilistic Reversal Learning Task (PRLT [28]). Some of these were however not relevant for the purposes of this study and will not be described here (see [42]; there is an overlap of 12.6% between samples of both studies).

### Instruments

The *South Oaks Gambling Screen* (*SOGS*, Spanish version; [37]) was originally based on DSM-III-R diagnostic criteria for pathological gambling, but it has been later shown to have good convergence with DSM-IV-TR and DSM-5 [43]. The clinical threshold for gambling disorder has been established at mean score ≥ 5. The Spanish version of this instrument has shown good psychometric properties (Cronbach’s α = 0.94 [37]).

*Alcohol and drug misuse* were assessed with the eight dichotomous alcohol and drug-related items of the *MultiCAGE* questionnaire (Spanish version; [41]). The Spanish version of this questionnaire has shown good internal consistency as measured by Cronbach’s α (i.e. all scales presented scores higher than 0.70 [41]). Risk of alcohol misuse was computed as the average response for the 4 alcohol-related items (0–1), and risk of drug misuse as the average for the 4 drug-related items (0–1) of the scale. For the two sub-scales, the threshold for significant risk of misuse has been established at two positive responses (mean score ≥ 0.5) [41].

The *Probabilistic Reversal Learning Task* (*PRLT*, [28]), is a computer-based decision-making task, in which the participants have to choose, in each trial, between two different stimuli (by mouse-clicking on one of them). The options consist of two squares of different colors, randomly shifting their positions. The task consists of four phases with 40 trials each. Within each phase, one of the options was “correct” and, when the participant chose it, a symbolic reward was given in most occasions (probabilistically). The other option was “incorrect”, and the participant was notified of the error after choosing it. Participants were rewarded with virtual points, and punished by subtracting points from their account. In this way, phases 1 and 3 were phases with the original contingency sign, and 2 and 4 as phases with reversed contingencies. In phases 1 and 2, the proportion of true/false feedback was 80/20%, whereas in Phases 3 and 4 was 70/30%. This degradation of contingency was introduced to increase uncertainty and thus to avoid close-to-perfect performance in the late phases of the task.

### Statistical analyses

The two groups (patients and controls) were first compared in relevant sociodemographics and clinical features using Bayesian Mann–Whitney tests, with default priors as implemented in open JASP software.

PRLT performance was coded trialwise. Each response in each trial was classified as correct (*R* = 1) if the colored square with the higher probability of reward (in the ongoing phase) was chosen, and incorrect (*R* = 0) if the color with the lower probability of reward was chosen.

The first PRLT analysis obeyed to a Phase (1, 2, 3, 4) × Trial (1–40) × Group (HC, Patients) design. Response was modeled as a binomial variable with a logit link, using Generalized Linear Mixed-effects Models (GLME), with the *glmer* function implemented in the *lme4* R software package [44]. This analysis is conceptually similar to a logistic regression, but includes both random and fixed-effects factors. In the present case, Phase and Trial were used as within-participant fixed factors, and Group as a between-participant fixed factor. Participant was considered as a random-effects factor, and Trial also as a random slope at the participant level (trial|participant). Additionally, in order to reduce the number of parameters in the model, Trial was treated as a quantitative variable, and was (natural)log-transformed in order to incorporate into the models the general principle that acquisition processes are curvilinear (in relation to Trial), and can thus be modelled as approximately linear in relation to Log-trial. Log-trial was zero-centered with the standard deviation as unit (so Log-trial was expressed in a − 3.16 to 1.07 scale). Standardization is just a linear transformation of the original variable, and is generally recommended for quantitative predictors in this type of models to prevent convergence problems. For presentation purposes, the scale in all figures was restituted to the 1–40 scale.

Main effects in this analysis were thus the ones of Log-trial, Phase, and Group, and the interactions among them. The effect of Phase was decomposed into three orthogonal contrasts [C1 (− 1, − 1, 1, 1), C2 (1, − 1, 1, − 1), and C3 (− 1, 1, 1, − 1)]. The contrast portraying evidence regarding learning inflexibility is C2, as it represents the differential performance in phases with reversed-sign contingencies (2nd and 4th), relative to phases with the original contingency sign (1st and 3rd). Global learning differences are however portrayed by Group and Group × Log-trial main effects.

In order to isolate the contribution of each main effect to model fitting, a saturated model was first fitted. This was contrasted against a simplified one without the Phase × Log-trial × Group interaction, using the Akaike Information Criterion (AIC) and a likelihood ratio test. If the simplified model did not lose fit, it was established as the reference model for further comparisons, and was further simplified by removing the two-way interactions one by one. The same procedure was repeated with marginal effects (Phase, Log-trial, and Group, with the restriction that a marginal effect cannot be removed if it is involved in any of the interactions left in the model in previous steps). Once the best-fitting model was identified, significance of each of the effects in the model was determined using the *z* statistic, with a *p* < 0.05 significance level.

A second analysis was aimed at assessing the contribution of SOGS gambling severity to PRLT (both regardless and controlling for group affiliation). A similar backwards hierarchical model fitting procedure was followed, but including SOGS Severity as an individual differences factor (instead of, or along with Group).

## Results

### Preliminary analyses

Each group consisted of 18 males and 7 females. Education years and Income were not available for one participant, and those two missing data points were imputed using group means. Mean and standard deviation for each group in Age, Education years, and the Monthly income scale are shown in Table 1.

Bayes factors (for the Mann–Whitney U test) yielded support for the alternative hypothesis (BF_10_ > 3) for Education years, and for the null (BF_10_ < 1/3) for Age and Monthly income. In other words, the two groups were well matched in Age and Monthly income, but differed in Education years. The same analyses were performed for the SOGS, MultiCAGE drugs, and MultiCAGE alcohol. As expected, given the group sampling procedure, patients were, on average, well above the clinical threshold in the three scales, whereas controls scored clearly within the non-problematic range. Complementarily, neither Monthly income (*r* = − 0.073, BF_10_ = 0.201), nor Education years (*r* = − 0.194, BF_10_ = 0.429) substantially correlated with SOGS severity across groups.

Before proceeding to the main analyses, we also checked whether log-transforming trial number within phase served the aim of adequately capturing the hypothetical curvilinear shape of the learning function (i.e. the learning process underlying the probability of a correct choice is a linear function of Log-trial), and the shape restrictions imposed by this assumption are not as tight as to not allowing to capture variations of learning curves across levels of the other factors. With that aim in mind, three baseline models were compared (identical to the ones described in the statistical analyses section, except for the non-inclusion of Group, and how Trial was modelled). In the first one (*linear*), trial was not transformed; in the second one (*logarithmic*) trial was (natural)log-transformed before entering the model; and in the third one (*polynomial*) the effect of trial was decomposed into a quadratic and linear component. Both the logarithmic and the polynomial model clearly outperformed the linear one (AIC = 10,509, 10,492, and 10,505, for the three models, respectively), which indicates that the learning process is better conceptualized as curvilinear function of trial. However, despite being more flexible (having less shape restrictions and thus being able to capture a broader range of curves) the polynomial model was outperformed by the logarithmic model. Or, what amounts to be the same, the increase in explained variance does not compensate the increase in complexity of the polynomial model (13 vs 9 parameters).^1^

**Fig. 1:**
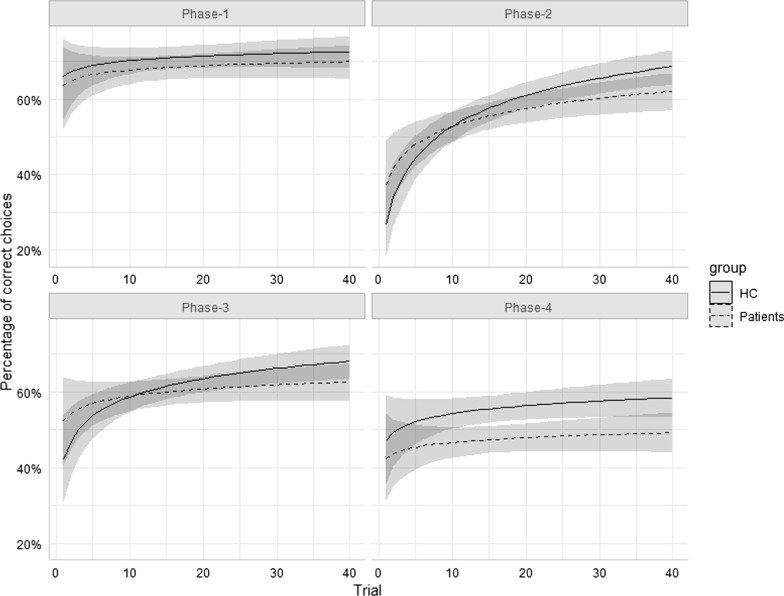
Predicted values (and confidence intervals) from the saturated model in Table 2, for controls (HC) and patients, across Phase and Log-trial. The vertical axis represents the predicted probability of a correct choice

**Fig. 2:**
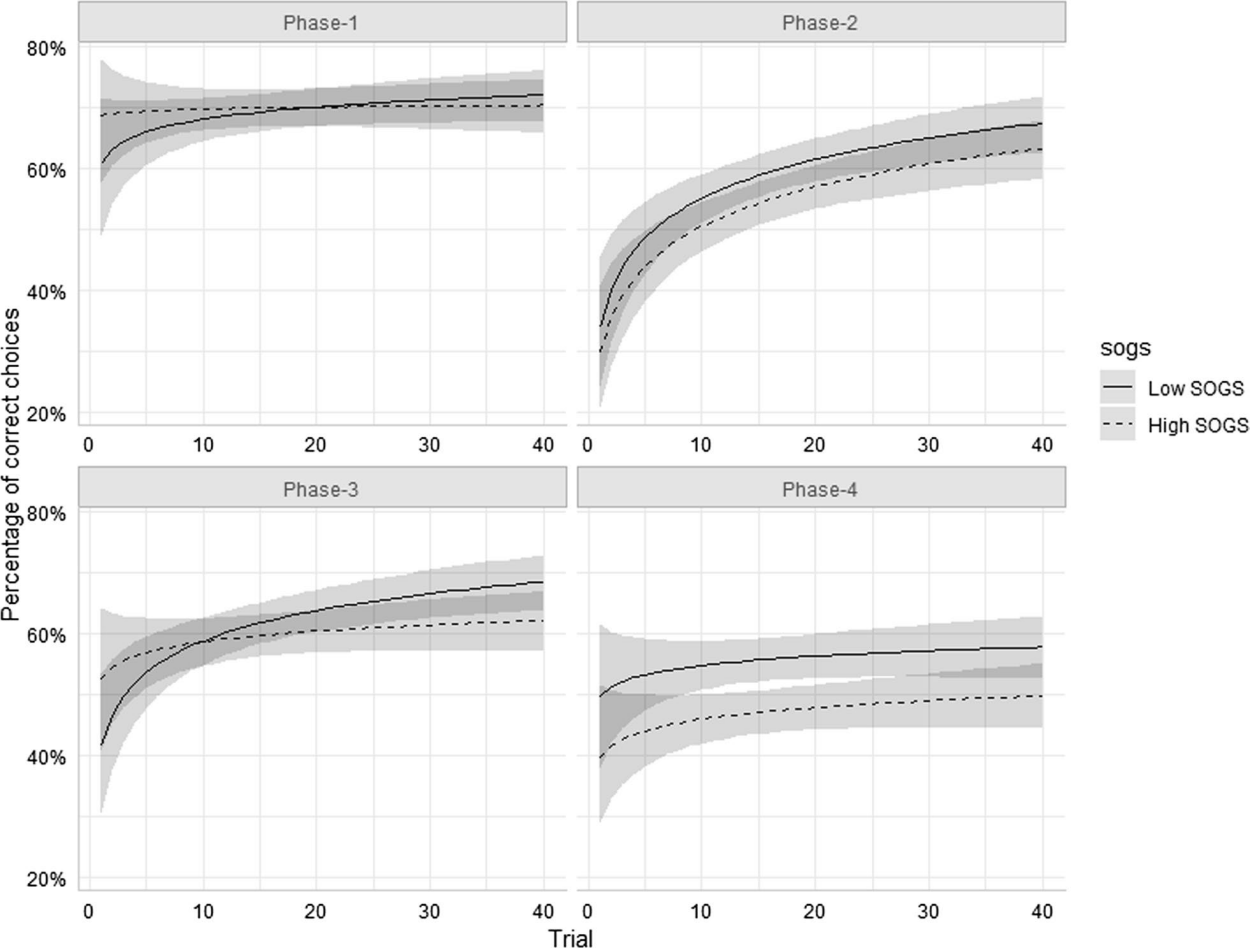
Predicted values (and prediction confidence intervals) for all Phase × Block conditions in the PRLT, for low and high SOGS level, from the saturated model in Table 4. SOGS reference values were automatically selected as high (+ 1 SD), and low (− 1 SD)

### Between-groups differences in PRLT performance

Table 2 shows results for the hierarchical GLME analysis. Removing the three-way interaction from the saturated model (Model 0.a vs Model 1) did not hamper model fit. Removing the Group × Phase interaction or the Group × Trail interaction did not affect model fit either (Model 2.1 and 2.3 vs Model 1). However, removing the Phase × Log-trial interaction from Model 1 (Model 2.2 vs Model 1) did hamper model fit, so that two-way interaction was retained. Further removal of Group from Model 2.4 did not hamper model fit either, so the final, best-fitting model (Model 3) did not contain any marginal or interactive effect of Group.

**Table 2:** Model selection for PRLT performance in the two-groups sample

Model	Fixed factors	df	*AIC*	χ^2^	*p*
Sat. (0.a)	Group, Phase, Log-trial, 2-way interactions, 3-way interaction	19	10,422		
1	Saturated minus 3-way interaction	16	10,419	2.418	0.490 (1 ≥ 0.a)
2.1	Model 1 minus Group × Log-trial	15	10,418	1.418	0.227 (2.1 ≥ 1)
2.2^b^	Model 1 minus Phase × Log-trial	13	10,432	18.978	* < 0.001* (1 > 2.2)
2.3	Model 1 minus Phase × Group	13	10,418	4.973	0.174 (2.3 ≥ 1)
2.4	Model 1 minus Group × Log-trial and Phase × Group	12	10,417		
3^a^	Model 2.4 minus Group	11	10,416	0.592	0.459 (3 ≥ 2.4)

Significant *p* values are in italics

^a^Best fitting model

^b^Almost singular fit (given the risk of overfitting, parameters will be estimated both for Model 1 and Model 3) (Although singular models are statistically well defined, singular fits may correspond to overfitted models with low power, and inferential procedures such as likelihood ratio tests may be inappropriate. In our case, singularity is due to the inclusion of Log-trial as a random slope in the model. Although it is theoretically sensible to assume that there are random individual differences in learning rates across participants, random slopes are not necessary to capture statistical dependency between repeated measures and thus to properly estimate within-participant effects. In view of that, and for the sake of consistency, alternative analyses without random slopes in the models are provided in the Additional file)

*Sat* Saturated

Table 3 presents estimates for all effects (*OR*, odds ratios) in models 1 and 3, along with their confidence intervals and significance levels, resulting from running the models. The directions of these effects are shown in Fig. 1. Predicted values from the saturated model show, that, as expected, the proportion of correct responses increased with Log-trial within phases; and, second, that performance in reversed phases decreased relative to acquisition-sign-contingency phases, regardless of Group. There was no significant evidence that patients made fewer correct choices in general, or in phases with reversed contingencies relative to non-reversed ones (and so the absence of Group marginal or interactive effects in the best-fitting model, and particularly of interactions involving Group × C2).

**Table 3:** Effect estimates for Model 1 and the best-fitting model (Model 3) of correct choices in the PRLT

Predictors	Model 1			Best-fitting model		
	*OR*	*CI*	*p*	*OR*	*CI*	*p*
Intercept	1.62	1.39–1.89	* < 0.001*	1.50	1.35–1.68	* < 0.001*
Log-trial	1.24	1.11–1.38	* < 0.001*	1.18	1.09–1.28	* < 0.001*
Phase C1	0.88	0.82–0.94	* < 0.001*	0.86	0.82–0.90	* < 0.001*
Phase C2	0.81	0.76–0.87	* < 0.001*	0.79	0.76–0.83	* < 0.001*
Phase C3	0.93	0.87–0.99	*0.021*	0.96	0.92–1.01	0.082
Group	0.86	0.69–1.07	0.165			
C1 × Log-trial	0.97	0.92–1.01	0.153	0.97	0.92–1.01	0.153
C2 × Log-trial	1.04	1.00–1.09	0.077	1.04	1.00–1.09	0.078
C3 × Log-trial	1.09	1.04–1.14	* < 0.001*	1.09	1.04–1.14	* < 0.001*
Group × Log-trial	0.91	0.78–1.06	0.224			
C1 × Group	0.95	0.87–1.05	0.326			
C2 × Group	0.95	0.86–1.04	0.232			
C3 × Group	1.08	0.98–1.18	0.123			
**Random effects**						
σ^2^	3.29					
τ_00_	0.13_Participant_					
τ_11_	0.05_Log-trial|Participant_					
ρ_01_	1.00					
ICC	0.05					
N	50					

Significant *p* values are in italics

### PRLT performance as a function of SOGS gambling severity

The patients group consisted of individuals receiving treatment for substance use disorders who also presented gambling problems. So, in order to test gambling problems in a more specific way, the impact of SOGS gambling severity on PRLT was analyzed. We did so by including SOGS score as a continuous predictor, along with its interactions with Phase and Trial, in the best-fitting model from the previous analyses, and reached the best-fitting model following the hierarchical procedure described earlier (Table 4, left panel). Although Group did not have any marginal or interactive effects in previous analysis, for the sake of robustness, effects were also estimated for SOGS effects while controlling for Group and Group × Phase (i.e. Group-related effects that could explain SOGS and SOGS × Phase effects away; Table 4, right panel).

**Table 4:** Model selection for PRLT performance, including SOGS severity

Model	Fixed factors	df	AIC	χ^2^	*p*		Model	Fixed factors	df	AIC	χ^2^	*p*
Sat. (0.b)	Model 3 plus SOGS and its interactions with Phase and Log-trial	19	10,418				Sat (0.c)	Model 3 plus Group and Group × Phase plus SOGS and its interactions with Phase and Log-trial	23	10,421		
4^b^	0.b minus SOGS × Phase × Log-trial	16	10,415	2.81	0.422 (4 ≥ 0.b)		6^b^	0.c minus SOGS × Phase × Log-trial	20	10,418	0.82	0.420 (6 ≥ 0.c)
5.1^a,b^	Model 4 minus SOGS × Log-trial	15	10,414	0.59	0.443 (5.1 ≥ 4)		7.1^a,b^	Model 6 minus SOGS × Log-trial	19	10,417	0.59	0.444 (7.1 ≥ 6)
5.2^b^	Model 4 minus Phase × Log-trial	13	10,428	19.05	* < 0.001* (4 > 5.2)		7.2^b^	Model 6 minus Phase × Log-trial	17	10,431	19.05	* < 0.001* (6 > 7.2)
5.3^b^	Model 4 minus Phase × SOGS	13	10,418	8.34	*0.039* (4 ≥ 5.3)		7.3^b^	Model 6 minus Phase × SOGS	17	10,421	8.43	*0.038* (6 ≥ 7.3)

Significant *p* values are in italics

^a^Best fitting model

^b^Almost-singular fit: given the risk of overfitting, parameters will be estimated in both best-fitting and saturated (0.b and 0.c) models. See also footnote 2

*Sat* Saturated

Interestingly, SOGS interaction with Phase involved only contrast C2 (see Table 5), namely the one reflecting learning inflexibility (reacquisition during phases with reversed contingencies, relative to the one in phases with the original contingency sign). The C2 contrast is significant in all models considered so far, i.e. learning is poorer in reversed contingency phases than in non-reversed ones. However, this pattern was more intense in high-SOGS individuals. As can be seen in Fig. 2, high-SOGS individuals showed a more intense drop in the probability of making a correct choice in Phase 2 relative to Phase 1, and in Phase 4 relative to Phase 3, if compared with low-SOGS.

**Table 5:** Effect estimates for saturated (0.c) 1 and best-fitting models (7.1) of correct choices in the PRLT (controlling for Group)

Fixed effects	Saturated model			Best-fitting model		
	*OR*	*CI*	*p*	*OR*	*CI*	*p*
Intercept	1.49	1.27–1.74	* < 0.001*	1.49	1.27–1.74	* < 0.001*
Log-trial	1.18	1.09–1.28	* < 0.001*	1.18	1.09–1.28	* < 0.001*
Phase C1	0.85	0.79–0.93	* < 0.001*	0.85	0.79–0.93	* < 0.001*
Phase C2	0.77	0.70–0.83	* < 0.001*	0.77	0.70–0.83	* < 0.001*
Phase C3	0.89	0.82–0.97	*0.007*	0.89	0.82–0.97	*0.007*
Group	1.02	0.82–1.27	0.849	1.02	0.82–1.27	0.849
SOGS	0.92	0.80–1.05	0.234	0.95	0.85–1.06	0.363
Log-trial × C1	0.97	0.92–1.01	0.158	0.97	0.92–1.01	0.153
Log-trial × C2	1.04	1.00–1.09	0.075	1.04	1.00–1.09	0.075
Log-trial × C3	1.09	1.04–1.14	* < 0.001*	1.09	1.04–1.14	* < 0.001*
Group × C1	1.01	0.88–1.16	0.870	1.01	0.88–1.16	0.869
Group × C2	1.07	0.93–1.23	0.337	1.07	0.93–1.23	0.342
Group × C3	1.16	1.01–1.33	*0.039*	1.16	1.01–1.33	*0.039*
Log-trial × SOGS	0.97	0.90–1.05	0.440			
SOGS × C1	0.96	0.90–1.03	0.258	0.96	0.90–1.03	0.261
SOGS × C2	0.92	0.86–0.99	*0.019*	0.92	0.86–0.99	*0.018*
SOGS × C3	0.95	0.89–1.02	0.160	0.95	0.89–1.02	0.163
Log-trial × SOGS × C1	0.99	0.95–1.04	0.776			
Log-trial × SOGS × C2	1.04	0.99–1.09	0.119			
Log-trial × SOGS × C3	0.99	0.94–1.04	0.651			
**Random effects**						
σ^2^	3.29					
τ_00_	0.13_Participant_					
τ_11_	0.05_Log-trial|Participant_					
ρ_01_	1.00					
ICC	0.05					
N	50					

Significant *p* values are in italics

## Discussion

The first aim of the present study was to test the existence of PRLT differences, and, more specifically, signs of decision-making inflexibility, in a group of patients with addictive disorders and gambling problems, relative to matched controls. As depicted in Fig. 1 (see Additional file 1: Figure S1 for a depiction of observed, instead of predicted, responses), although patients showed less steep within-phase learning functions, between-group differences did not reach significance.^2^ There were no specific learning efficiency drops either in phases with reversed contingencies (phases 2 and 4), relative to non-reversed ones (phases 1 and 3). Some studies have found that addiction may have a generalized deleterious effect on feedback-based decision-making [27, 28], but this does not make learning necessarily more inflexible.

Results were contrastingly different when PRLT performance was analyzed as a function of gambling severity (measured with the SOGS questionnaire). As shown in Fig. 2 (and Additional file 1: Figure S2), participants with stronger disordered gambling symptoms made substantially fewer correct choices in phases 2 and 4, relative to phases 1 and 3, regardless of group. Or, more precisely, the more severe gambling was, the more marked this pattern grew. This result also aligns with the ones from the study by Torres et al. [29], in which gambling intensity (monthly use) was associated with increased reversal costs, restricted to reversed-contingency phases, and, especially, with Perandrés-Gómez et al.’s, [21] in which patients with gambling disorder were found to behave more inflexibly in the PRLT, independently of drug use. To our knowledge, the only study in which this pattern has been reported in patients with substance use disorders is the one by Moreno-López et al. [28], where reversal learning deficits were observed to be associated with cocaine use severity and diminished cerebellar gray matter volume.

In summary, according to our results, restricted effects on reversal deficits (namely, specific difficulties in learning reversed contingencies), can arise independently of differences in general learning deficiencies in the PRLT. As shown here, only the former seem to be associated with degrees of gambling severity; value updating of choice options seems to be more hindered in more severe gamblers.

Beyond this effect, this work also presents some methodological advances. So far, PRLT performance had been assessed either by extracting summary performance indices (e.g. number of perseverative errors; [30, 31]) or analyzing learning curves in a blockwise fashion (number of correct responses per 5-trial or 10-trial block: [28, 29]). These summary parameters, however, present interpretation problems. For instance, individuals reaching higher learning asymptotes in the preceding phase tend to perform transitorily worse in the first trials of the ongoing phase. This means that assessing decision-making inflexibility by means of perseverative errors is likely to confound ‘true’ and ‘apparent’ perseverative errors, with the latter being attributable to pre-reversal differences. Blockwise analyses, in turn, are likely to be insensitive to effects that occur in the trial-by-trial scale. Moreover, our trialwise analyses of responses allows to model them as they really are, dichotomous (0/1) responses, instead of response counts (number of perseverative errors, number of correct choices per block) with distributional features that are seldomly taken into account in standard, general linear model-based analyses.

Taken together, results fit well in the *Gambling Space Model* formulated by Navas et al. [45] (see also [42, 46]). In this model, articulated as a development of the seminal *Pathways Model* [47], transition from recreational to disordered gambling is driven by the kind of reinforcement schedules that have been experimentally shown to also facilitate transition from goal-driven to compulsive behaviors. This transition towards ‘gambling-specific’ compulsivity can be speeded or made more likely in vulnerable individuals showing trait-like signs of compulsivity (as also shown by animal translational research; [48]). In view that in gambling disorder there is no chemical agent to hijack reinforcement circuits, individual differences in compulsivity proneness could play a larger role than in substance use disorders. Tentatively, this could explain why signs of compulsivity are easier to detect in patients with gambling disorder than in other populations of addicted individuals, and also why, in the present study, decision-making inflexibility did not emerge in the between-group comparison, but did when gambling severity was specifically taken into consideration.

As noted in the introduction, modelling inflexibility in computational terms is beyond our current aims. However, inflexibility, as operationalized here, arises as a behavioral pattern these models must be able to accommodate. For instance, the experience-weighted attraction model [EWA; 49, 50], includes an experience weight parameter (ρ) to capture the well-known fact that updating becomes slower as experience accumulates. Our finding that high-SOGS individuals are more prone to persevere in the phase of negative feedback (during reversed phases) is likely to be accounted for by an increased experience decay factor, as opposed to more aspects of reward learning (i.e. learning rate, α). Alternatively, our and Perandrés-Gómez et al.’s [21] results may be explained by differences in more complex (sequential) exploratory behavior as captured by another recent model [VSE; 51]. An example of how parameters included in computational models, estimated from behavior in decision-making-under-ambiguity tasks (i.e. the Iowa Gambling Task), can be used as individual differences variables to predict clinically-relevant gambling behavior can be found in a recent work by Kildahl et al. [52].

## Limitations and final remarks

The present work is not free of limitations. First, convenience sampling did not allow carrying out an a priori power analysis. Although taking all observations into account increases power (relative to, for example, block-wise analyses), 25 participants per group are probably still insufficient to reach adequate power for all relevant effects.

Second, the cross-sectional nature of the study does not allow either to make strong claims about causal directionality. More specifically, the fact that inflexibility is associated with gambling severity is no direct proof that gambling severity and inflexibility are causally related in one way or the other.

And third, and relatedly, controlling for group (that is, for the diagnosis of a substance use disorder) when testing SOGS-inflexibility association does not stand as a strong control of other potentially relevant variables as, for example, cognitive deterioration or drug use beyond the established diagnosis. Although MultiCAGE measures are available for all participants, this questionnaire is a screening test, and thus not adequate as a continuous measure of addiction severity.

In summary, the evidential value of the present findings must be assessed in combination with previously reported signs of proneness to learning inflexibility (i.e. domain-general compulsivity) in patients with disordered gambling. Seemingly, inconsistency in previous research can be attributed to differences in the way in which learning inflexibility in the Probabilistic Reversal Learning Task (PRLT) is operationalized. In the present study, we adopted a mostly-data driven approach to identify specific signs of learning inflexibility (anomalies restricted to reacquisition in phases with a reversed contingency sign). Only patients with more severe symptoms of problematic gambling showed specific signs of learning inflexibility. These results align with gambling disorder models in which domain-general compulsivity is linked to vulnerability to develop gambling-specific problems with exposure to gambling opportunities.

## Supplementary information

**Additional file 1.** Supplementary analyses.

## Data Availability

The open database and code files for these analyses are available without restriction at the Open Science Framework website (https://osf.io/qbejc/).

## Abbreviations

AIC: Akaike Information Criterion
CRIAD: Centro de Recuperación Nueva Luz and Centro de Recuperación Integral de Alcoholismo y Drogadicción
DSM-V: Diagnostic and Statistical Manual of Mental Disorders, Fifth Edition
DSM-IV: Diagnostic and Statistical Manual of Mental Disorders, Fourth Edition
EWA: Experience-weighted attraction model
GLME: Generalized Linear Mixed-effects Models
HC: Healthy controls
PRLT: Probabilistic Reversal Learning Task
Sat: Saturated
SOGS: South Oaks Gambling Screen
SPSRQ-20: Sensitivity to Punishment and Sensitivity to Reward Questionnaire-20 items
UPPS-P: Urgency, Premeditation, Perseverance, Sensation seeking, and Positive urgency Impulsive Behavior Scale
VSE: Value plus Sequential Exploration model
WAIS-III: Wechsler Adult Intelligence Scale, Third Edition

## Footnotes

1 The capacity of the log-transformation of the Trial scale to capture the shape of learning functions is visually corroborated by the close correspondence between Figs. 1 and 2 (predicted scores from fitted models) and Additional file 1: Figures S1 and S2 (observed scores).

2 This lack of significance is however likely to be due low power. Alternative analyses without the Log-trial random slope, presented in the first section of the Supplementary materials, yielded a significant Group × Log-trial effect. This effect originated in the generally slower learning functions in all Phases for the Patients group.
