# Supplementary material for: Decision-making inflexibility in a reversal learning task is associated with severity of problem gambling symptoms but not with a diagnosis of substance use disorder
Source: BMC Psychol. 2020 Nov 10;8:120. doi: 10.1186/s40359-020-00482-6 (PMC7654010; doi:10.1186/s40359-020-00482-6)
Supplement: Supplementary file 1 — Additional file 1. Supplementary analyses. [file 40359_2020_482_MOESM1_ESM.doc]

**Additional file 1**

**Random-intercept analyses**

As noted in the main text, models with random intercepts and slopes per participant resulted in singular fit in some cases. Removing the random slopes can be justified on the basis that a) changes in the effect of Log-trial across phases and groups is captured by the Log-trial x Group, the Log-trial x Phase, and the Log-trial x Group x Phase interactions; and b) the inclusion of participant intercepts captures statistical dependency between repeated measures. Not including the random slopes implies the assumption that any variability in the observed measures not accounted for by the fixed and random-effects predictors in the model are attributable to a common, normally-distributed error term (σ^2^), which is independent of any predictors in the model. In view of this, we run supplementary analyses without the Log-trial random slope, in all other senses identical to the ones in the main text.

Additional file 1: Table S1 shows results for the hierarchical GLME analysis of Group-related effects. Removing the three-way interaction from the saturated model (A0 vs A1) did not hamper model fit. However, removing either the Phase x Log-trial or the Group x Log-trial interaction from Model A1 (A1 vs A2.1, and A1 vs A2.2) did reduce model fit, so those two-way interactions were retained in the best-fitting model. Removing the Group x Phase interaction did not affect model fit (*p* = 0.188), and the marginal effects cannot be removed because they are involved in the two-way interactions previously retained in the model.

In view of the difference between groups in Education years shown in preliminary analyses, these models were re-run after including Education years and its interaction with Log-trial and Phase. These predictors did not substantially contribute to model fit, nor changed the results regarding any other predictors.

Additional file 1: **Table S1.** Model selection for PRLT performance in the two-groups sample.

| **Model** | **Fixed factors** | **df** | **AIC** | **χ^2^** | ***p*** |
| --- | --- | --- | --- | --- | --- |
| *Saturated (A0.a)* | Group, Phase, Log-trial, 2-way interactions, 3-way interaction | 17 | 10494 |  |  |
| *A1* | Saturated (A0.a) *minus* 3-way interaction | 14 | 10491 | 2.463 | 0.482  (A1 ≥ A0.a) |
| *A2.1* | A1 *minus* Group x Log-trial | 13 | 10493 | 4.249 | **0.039**  (A1 > A2.1) |
| *A2.2* | A1 *minus* Phase x Log-trial | 11 | 10504 | 19.43 | **< 0.001**  (A1 > A2.1) |
| *Best-fitting (A2.3)* | A1 *minus* Phase x Group | 11 | 10490 |  |  |
| Note: All models include a random intercept for participant. | | | | | |

Notably, and at difference with the analyses shown in the main text, Group interacted with Log-trial. This effect was estimated as OR = 0.91 [CI (0.83 – 0.99)], indicating a slightly slower acquisition in all phases for the group of patients. [Note that this effect was estimated as OR = 0.91, CI (0.71 – 1.06), in the analysis in the main text, so effect size’s point-estimates in the two analyses are actually coincident], This worsening did not significantly involve learning inflexibility, i.e. differences were not significantly larger in phases with reversed contingencies for patients, as shown by the absence of a Group x Phase interaction [OR = 0.95, CI (0.86 – 1.04), for the Group x C2 interaction in the A1 model from Additional file 1: Table S1].

In summary, results differ across analyses in a substantial aspect, but this difference does not change conclusions regarding inflexibility. Whether or not patients performed globally worse than controls in the PRLT is a matter of theoretical importance (see discussion in the main text), but our hypotheses exclusively regarded local decreases in reacquisition, after controlling for global acquisition effects. Regardless of whether this global effect is significant or not, patients did not behave more inflexibly in the PRLT.

Subsequently, SOGS was included in the best-fitting model resulting from the previous step. Additional file 1: Table S2 shows the result of the corresponding hierarchical analysis.

Additional file 1: **Table S2.** Model selection for PRLT performance in the two-groups sample, including SOGS.

| **Model** | **Fixed factors** | **df** | **AIC** | **χ^2^** | ***p*** |
| --- | --- | --- | --- | --- | --- |
| *Saturated (A0.b)* | Best-fitting model from the previous analysis *plus* SOGS and its interaction with Log-trial and Phase | 19 | 10494 |  |  |
| *A3* | Saturated (A0.b *minus* 3-way interaction | 16 | 10491 | 2.890 | 0.409  (A3 ≥ A0.b) |
| *A4.1* | A3 *minus* Phase x Log-trial | 13 | 10504 | 19.536 | **< 0.001**  (A3 > A4.1) |
| *A4.2* | A3 *minus* Phase x SOGS | 13 | 10493 | 8.313 | **0.040**  (A3 > A4.2) |
| *Best-fitting (A4.3)* | A3 *minus* Log-trial x SOGS | 15 | 10489 |  |  |
| Note: All models include a random intercept for participant. | | | | | |

Results from these analyses are thus qualitatively identical to the ones in the main text. The theoretically key SOGS x C2 contrast (from the SOGS x Phase main effect) was estimated at OR = 0.94, CI (0.90 – 0.99), p = 0.013. Interestingly, if the Group x Phase effect was retained in the initial model for further comparisons involving SOGS, the C2 x Group effect completely vanished [OR = 1.07, CI (0.93 – 1.23), p = 0.331], whereas the C2 x SOGS effect survived [OR = 0.92, CI (0.86 – 0.99), p = 0.017].

**Observed responses across Group levels**

**Additional file 1: Figure S1.** Observed proportion of correct responses for each phase of the PRLT task, for Patients and Controls (HC).

| Phase 1  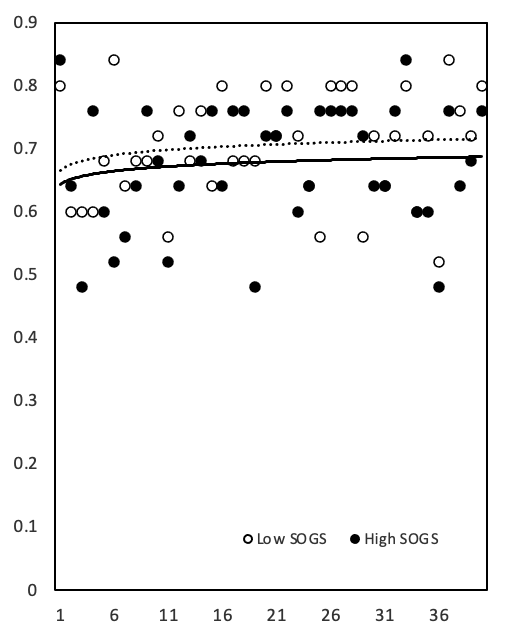 | Phase 2  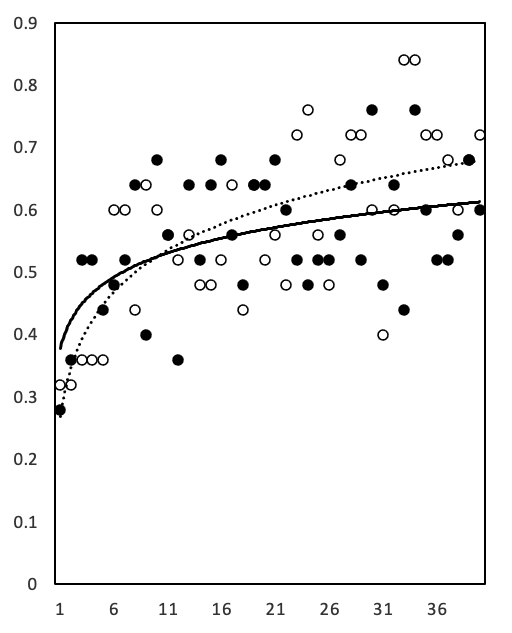 |
| --- | --- |
| Phase 3  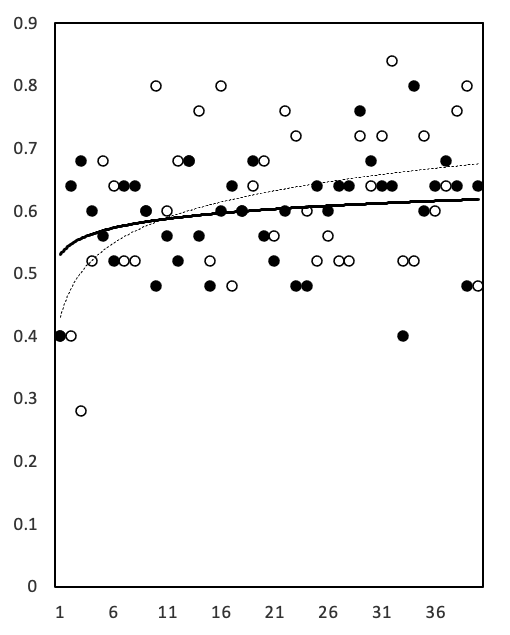 | Phase 4  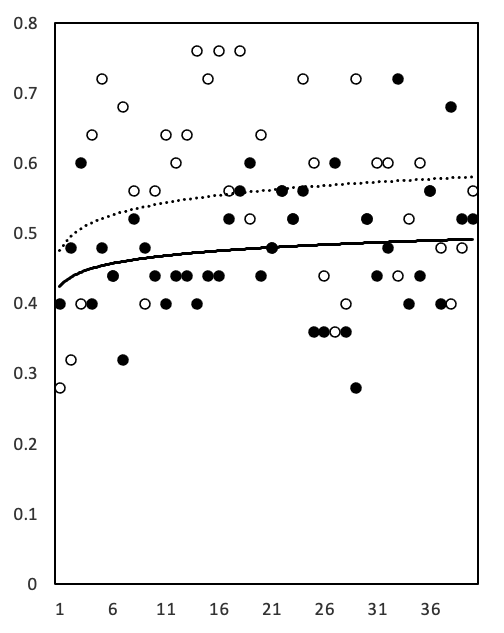 |

**Observed responses across SOGS values**

**Additional file 1: Figure S2.** Observed proportion of correct responses for low-SOGS participants (Mean SOGS - SD/2) and high-SOGS participants (Mean SOGS + SD/2).

| Phase 1  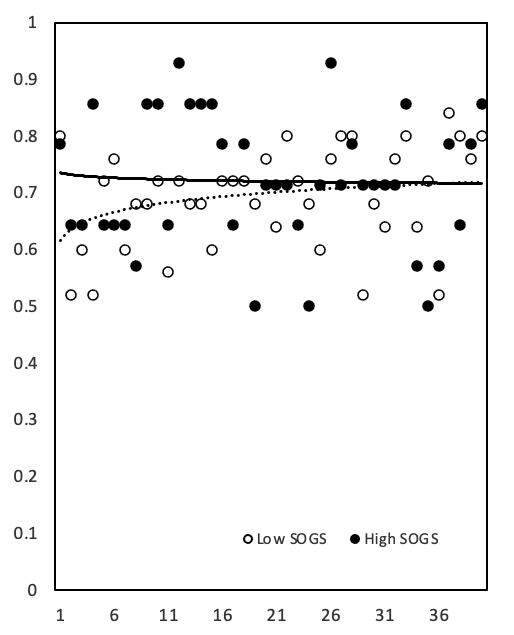 | Phase 2  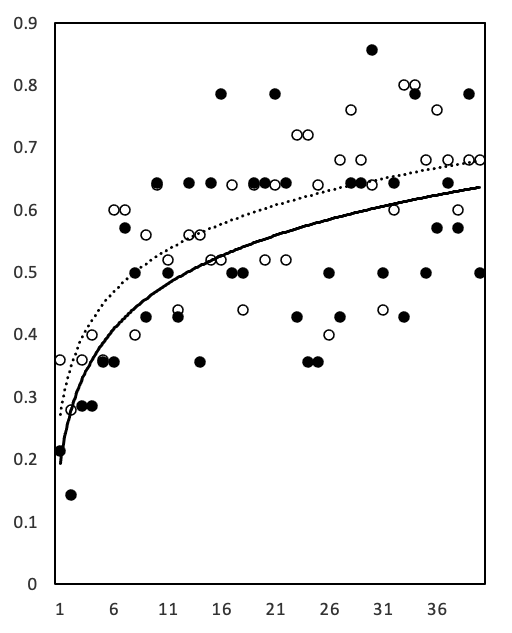 |
| --- | --- |
| Phase 3  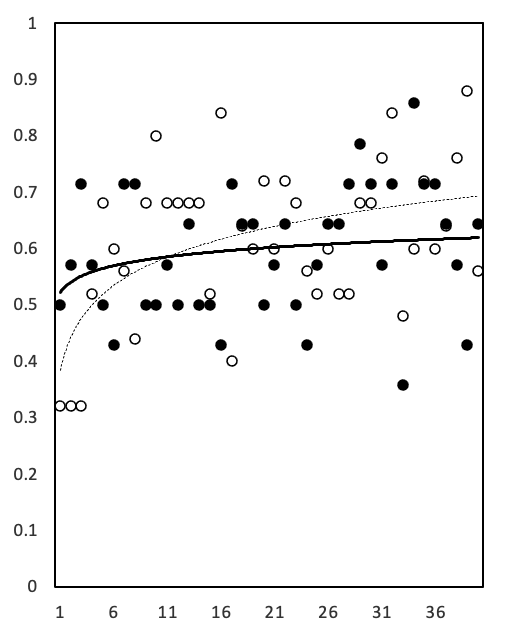 | Phase 4  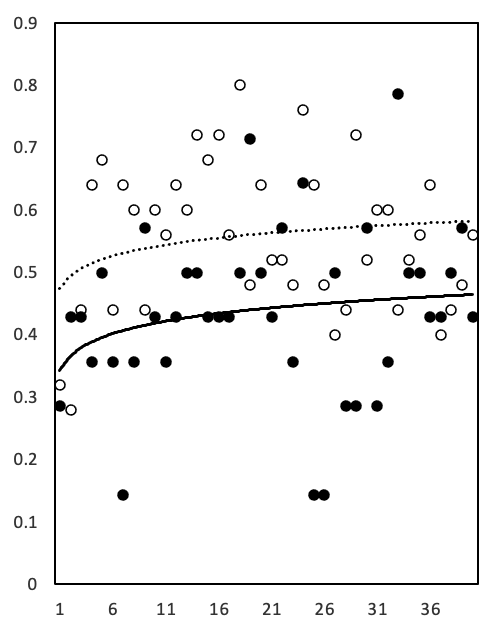 |
